# PAK1 and Therapy Resistance in Melanoma

**DOI:** 10.3390/cells12192373

**Published:** 2023-09-28

**Authors:** Julia V. Kichina, Alexei Maslov, Eugene S. Kandel

**Affiliations:** 1 Department of Immunology, Roswell Park Comprehensive Cancer Center, Elm & Carlton St., Buffalo, NY 14263, USA; 2 Department of Cell Stress Biology, Roswell Park Comprehensive Cancer Center, Elm & Carlton St., Buffalo, NY 14263, USA

**Keywords:** chemotherapy, immune therapy, targeted therapy, cutaneous melanoma, acral melanoma, uveal melanoma, PAK1, RAC1

## Abstract

Malignant melanoma claims more lives than any other skin malignancy. While primary melanomas are usually cured via surgical excision, the metastatic form of the disease portents a poor prognosis. Decades of intense research has yielded an extensive armamentarium of anti-melanoma therapies, ranging from genotoxic chemo- and radiotherapies to targeted interventions in specific signaling pathways and immune functions. Unfortunately, even the most up-to-date embodiments of these therapies are not curative for the majority of metastatic melanoma patients, and the need to improve their efficacy is widely recognized. Here, we review the reports that implicate p21-regulated kinase 1 (PAK1) and PAK1-related pathways in the response of melanoma to various therapeutic modalities. Ample data suggest that PAK1 may decrease cell sensitivity to programmed cell death, provide additional stimulation to growth-promoting molecular pathways, and contribute to the creation of an immunosuppressive tumor microenvironment. Accordingly, there is mounting evidence that the concomitant inhibition of PAK1 enhances the potency of various anti-melanoma regimens. Overall, the available information suggests that a safe and effective inhibition of PAK1-dependent molecular processes would enhance the potency of the currently available anti-melanoma treatments, although considerable challenges in implementing such strategies still exist.

## 1. The Therapy of Metastatic Melanoma: An Unmet Need Remains despite the Recent Progress

Melanoma is a cancer originating from melanocytes. The most prevalent variety of melanoma (cutaneous melanoma) originates from the pigment-producing cells in the epidermis. Cutaneous melanoma is the deadliest skin malignancy: the American Cancer Society estimates the yearly death toll of this disease to be at nearly eight thousand people in the USA alone [1]. While early-stage cutaneous melanoma is curable in >99% of cases, it is also known to metastasize relatively early, and fewer than a third of the patients with distal metastases survive for 5 years after diagnosis [1]. Melanomas may also arise from melanocytes at other locations, albeit with a much lower incidence. An example of that is a relatively rare uveal melanoma, which, nevertheless, is the most common type of eye cancer in adults. Uveal melanoma is commonly diagnosed when it is relatively large [2]. In this disease, the overall long-term rate of metastasis exceeds 60% [3], and the outcomes for patients who present with metastatic disease are dismal [4]. The difficulty of properly diagnosing melanomas at uncommon sites or in uncommon patient populations (e.g., children) presents additional challenges to effective management of the primary disease before the onset of metastasis [5].

The treatment of metastatic melanoma necessitates strategies beyond surgical removal. Unfortunately, historically, melanoma has proved to be notoriously resistant to conventional chemotherapy, with low response rates and a rapid emergence of therapeutic resistance. The response rates to various chemotherapeutic regimens generally remained below 20% [6,7], with severe adverse effects being relatively common and the overall survival benefits remaining uncertain [8]. The five-year survival percentage among such treated patients did not exceed single digits [9]. 

A better understanding of the underlying oncogenic drivers in melanoma lead to the advent of targeted therapies [10,11,12], which, to date, have shown the best results in BRAF-driven cases. However, an effective targeted therapy is still lacking for a large cohort of cases driven by other oncogenic events, while even the BRAF-driven cases commonly develop resistance in the course of treatment. 

Encouragingly, cutaneous melanoma is one of the most immunogenic tumors, which made it an attractive target for immunotherapy [13,14]. A direct comparison of immunotherapy to genotoxic chemotherapy reveals that the former yields superior long-term outcomes, including in BRAF wild-type cases, where common targeted therapies would be inapplicable [15]. Indeed, over a third of patients with advanced disease can reach the 5-year survival milestone when treated with individual immune checkpoint inhibitors [16,17], and this could be increased to about half of the patients when the inhibitors are used in combination [17]. The long-term survival of some patients indicates that this approach can be curative. However, the efficacy of these drugs comes at the cost of relatively common severe adverse effects, while most of the patients still progress while under treatment. Furthermore, the efficacy of checkpoint inhibitors in uveal melanoma is much lower than in the cutaneous disease [18,19]. Most recently, tebentafusp, an ImmTAC (Immune mobilizing monoclonal T-cell receptor Against Cancer) demonstrated a statistically significant benefit in uveal melanoma [20]. However, this treatment is limited to HLA-A*02:01-positive patients and yields a rather modest increase in median survival: overall by <6 months, progression-free by <0.5 month, with a ~9% response rate [20]. 

**Figure 1:**
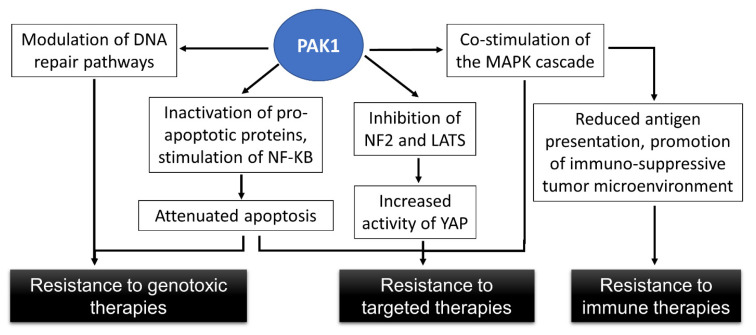
Proposed mechanisms linking PAK1 activity and melanoma resistance to various therapeutic modalities. See text for details.

Overall, despite the veritable progress in the management of melanoma, it is obvious that the currently available treatments for metastatic melanoma leave a lot of room for improvement. A better understanding of the molecular mechanisms of sensitivity and resistance to the currently available therapies leads to the identification of potentially druggable factors that may serve as intervention points to improve the efficacy of these treatments. One such factor, implicated in cancer responses to multiple treatment modalities, is p21^RAC1^-activated kinase 1 (PAK1) (Figure 1).

## 2. PAK1 Structure and Expression in Melanoma

PAK1 is a serine-threonine kinase conserved among eukaryotes as diverse as yeast, mammals, and flowering plants. Mammalian PAK1 was originally identified as a kinase, activated in the brain by Rho-family GTPases [21]. Subsequent research revealed that several homologous kinases are encoded by human and mouse genomes. Currently, this enzyme family consists of PAK1, PAK2, PAK3, PAK4, PAK5, and PAK6. Based on the degree of similarity within the family, the first three of these proteins are classified as Group I PAKs, while the rest—Group II. 

**Figure 2:**
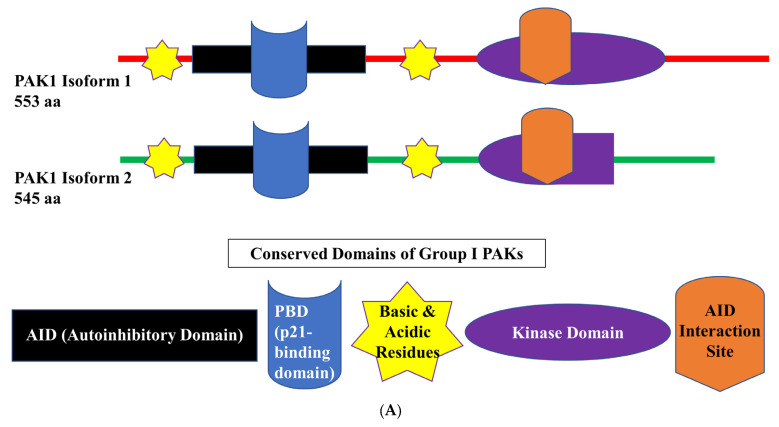

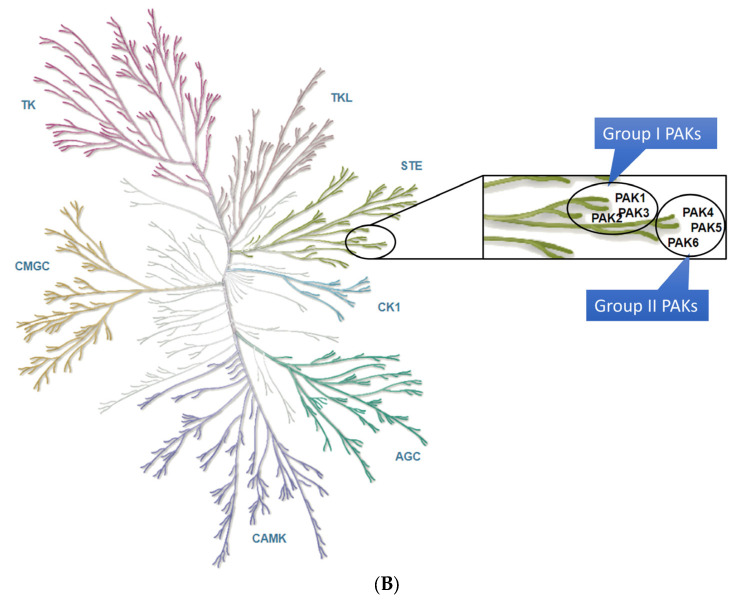
PAK1 structure and position within human kinome. (**A**) Conserved elements within PAK1 isoforms 1 and 2. (**B**) Position of PAK proteins on the evolutionary tree of human kinases. The evolutionary tree of kinases was drawn using KinMap [31].

The human PAK1 gene occupies over 150 kbps on the longer arm of chromosome 11. NCBI GenBank (https://www.ncbi.nlm.nih.gov/gene/5058; accessed on 19 July 2023) currently holds several dozen different transcripts of this gene, with this diversity being attributable to alternative splicing and alternative transcription initiation. Not all of these transcripts are equally well supported by experimental evidence, and some encode identical proteins despite the differences in the non-coding regions. Among the different encoded proteins, Isoform 1, consisting of 553 amino acids (Figure 2A), is the most commonly studied human variant of human PAK1. Several shorter isoforms of this protein have been reported as well. There is evidence that PAK1 isoforms may be functionally distinct [22,23] and that in melanoma, the predominant splicing pattern favors the creation of an enzymatically active PAK1 over a product of alternative splicing that encodes a truncated kinase domain (Isoform 2; Figure 2A) [24]. There is also some evidence that additional variation among the PAK1 transcripts may be introduced via alternative polyadenylation [25]. This potentially could change the susceptibility of PAK1 expression to the control of miRNAs [26]. Overall, the significance and mechanisms of the generation of the alternative PAK1 mRNAs, as well as the biological functions of the individual protein isoforms, still remain as areas in need of further exploration.

Based primarily on the structure of its kinase domain, human PAK1 is classified together with other PAKs within the STE20 family from the STE group of kinases [27] (Figure 2B). In addition to the kinase domain, PAK1 protein includes a p21^RAC^-binding domain (PBD), which overlaps with an auto-inhibitory domain (AID), as well as several conserved binding sites for adaptor proteins and guanine nucleotide exchange factors [28,29] (Figure 2A). Immediately preceding the PBD is a stretch of basic amino acids, which allows PAK1 to bind to certain lipids [30]. 

In its inactive state, PAK1 is a “head-to-tail” dimer, where the AIDs are bound to and inhibit the kinase domains [29]. The best-known mode of PAK1 regulation is its activation by the small GTPases of the Rho family, such as p21^RAC1^, from which the name of PAK1 is derived. These small GTPases gain affinity to PAK1 while bound to GTP [32], and this interaction relieves the kinase domain from the AID. This, in turn, facilitates the formation of a “face-to-face” dimer, where the monomers cross-phosphorylate each other, thus achieving full activation [33,34]. Importantly, constitutively active mutants of these GTPases demonstrate an enhanced affinity to PAK1 [35]. This mode of regulation also implies that other potential interactors of the autoinhibitory domain that diminish its affinity for the kinase domain would act as activators of PAK1. The interaction of PAK1 with certain lipids may also contribute to its activation, as well as to membrane localization [30,36].

Activated PAK1 is known to phosphorylate a great number of targets. While their complete inventory is beyond the scope of this review, the numerous biochemical and biological consequences of PAK1 activity have been extensively reviewed elsewhere [28,37,38,39,40]. The involvement of many of the PAK1-regulated factors in such phenomena as cell motility, cell proliferation, and survival support the continuous interest in PAK1 among cancer researchers and has led to the recognition of this enzyme as a bona fide target for anti-cancer therapy [28]. Indeed, there is abundant evidence of PAK1 being essential for the survival and proliferation of at least some cancer types, and this dependence correlates with the nature of oncogenic drivers in these malignancies. Melanomas commonly display elevated levels of PAK1 expression as well as its activity, with the latter being judged by the abundance of the appropriately phosphorylated form [41,42,43]. As mentioned above, PAK1 mRNA splicing in melanoma is skewed towards producing the kinase-proficient isoform of the protein [24]. The PAK1 gene is amplified in 5–9% of cutaneous melanomas [41,43]. The incidence is higher in acral melanomas [44,45], which also tend to retain wild-type BRAF and portent worse outcomes [46]. Importantly, the amplicon often includes other cancer-relevant genes (e.g., CCND1 and GAB2), which might further cooperate with PAK1 in oncogenesis. Nevertheless, gene amplification may explain only a fraction of the cases of PAK1 overexpression, and additional mechanisms that account for higher PAK1 levels are yet to be firmly established. The hyperactivation of PAK1 in cutaneous melanomas may be further attributed to activating mutations in RAC1 [47,48], but also to even more common activating mutations in NRAS and the inactivation of RAS inhibitor NF1 [47], as RAS-induced transformation involves and depends on the activation of RAC1 and PAK1 [49,50,51,52]. Activating mutations in KRAS and HRAS are also expected to activate PAK1, although these mutations are present in less than 2% of melanomas each [53]. Frequent mutations in PREX2 [54] which increase its capacity to act as a guanine nucleotide exchange factor (GEF) for RAC1 [55] are also expected to upregulate PAK1. Mutations in the KIT gene, encoding receptor tyrosine kinase c-KIT, are typically found in acral and mucosal melanomas [56,57] and may be predicted to activate a PAK1-mediated oncogenic pathway, as was documented in other malignancies [58]. The co-occurrence of several mutations that are potentially capable of activating PAK1 is not uncommon in cutaneous melanoma [59]. In a small subset of cutaneous melanomas, and in the vast majority of uveal melanomas, the activation of PAK1 is expected as a consequence of activating mutations in the GNAQ and GNA11 oncogenes, which reportedly signal through RAC1 and RAS [60,61]. Finally, there are intriguing observations that BRAF can directly activate PAK1 in the context of thyroid cancer [62]. If confirmed in the context of cutaneous melanoma, this phenomenon might be of great importance due to the high prevalence of BRAF mutations in this disease.

## 3. PAK1 and Genotoxic Therapy in Melanoma

In general, melanoma is relatively resistant to genotoxic chemo- and radiotherapy. Various conventional genotoxic chemotherapeutic regimens, primarily based on the alkylating agent dacarbazine, are known to achieve response in ~20% of metastatic cutaneous melanoma cases, with responses usually lasting for several months [9,63]. Prior attempts to improve the efficacy of such regimens by combining multiple genotoxic agents failed to yield a significant clinical benefit despite noticeably increasing the incidence of adverse effects [63]. 

Radiation therapy in melanoma is used to manage some primary tumors with limited surgical options, with acceptable long-term control in most lentigo maligna and uveal melanomas [64,65], although with less encouraging results in the mucosal form of the disease [66]. As an adjuvant for lymphadenectomy, radiation therapy offers short-term benefits, but does not improve the overall survival [67]. Radiation therapy is also commonly used for metastatic melanoma, where the effect is often palliative, but hardly ever curative [68]. An obvious drawback of radiation therapy is the damage to normal tissues, including vital organs [69].

There is sufficient evidence to implicate PAK1 in the response of cancer cells to genotoxic impacts, including those delivered in the course of therapy. Reportedly, PAK1 can be activated by at least some types of DNA damage [70]. Various lines of evidence point to the role of PAK1 in DNA repair [71]. A comparison between PAK1 wild-type and knockout cells following ionizing radiation suggested that PAK1 affects a wide array of genes and proteins with known roles in the DNA repair and DNA damage responses [72]. Those experiments were conducted on a single pair of mouse embryonic fibroblast cell lines, so their direct relevance to human melanoma cannot be taken for granted. Importantly, it was also reported that PAK1 can suppress the sensing of DNA damage and increase the tolerance of melanoma cells to genotoxic treatment [73]. In particular, PAK1 appears to attenuate p53 activation in human melanoma cells with wild-type p53 [73], and this parallels the de-repression of some of the p53 targets observed in PAK1-deficient mouse fibroblasts [72].

These observations add to the list of several previously reported avenues through which PAK1 may affect the pathway of programmed cell death: the inactivation of pro-apoptotic proteins BIM [74], BAD [75,76], and FOXO1 [77], and the stimulation of a pro-survival transcription factor, NF-κB [78]. Notably, some of the NF-κB targets are secreted factors that provide additional signals to further activate NF-κB [79]. Therefore, some of the consequences of PAK1 activation may not be cell-autonomous. Admittedly, these connections between PAK1 and the apoptotic machinery have been predominantly investigated in non-melanoma models, and their relevance to life/death decisions in melanoma cells is yet to be rigorously established. Nevertheless, the presence of the same factors and the corresponding pathways in melanoma cells makes it possible that these observations are relevant to melanoma as well.

The drive to increase the efficacy of conventional genotoxic therapy for melanoma has been somewhat obscured by the successes of other treatment modalities. However, the frequent eventual failure of the latter leaves unmet the need for effective care for heavily pre-treated recurring disease. In this context, there may be an opportunity to exploit PAK1 inhibition in conjunction with chemo- or radiotherapy to improve the plight of this category of patients. 

## 4. PAK1 and Targeted Therapies in Melanoma

PAK1 is positioned amidst multiple intracellular signaling pathways, including the ones that control cancer-relevant traits such as proliferation, resistance to apoptosis, invasiveness, motility, etc. [28]. In the context of melanoma, the so-called MAPK cascade is of a particular importance. This cascade normally transmits and amplifies the signal from the plasma membrane to intracellular components, including the nucleus. The pathway not only promotes cell growth and survival, but also imparts widespread changes in cell metabolism [80]. In the canonical scheme, the engagement of a receptor tyrosine kinase eventually leads to the activation of an RAS family protein, which in turn recruits to the plasma membrane and activates an RAF family member, which phosphorylates a kinase of the MEK family, whose targets are the MAP kinases, including those known as ERKs [81]. There are numerous variations on this theme, with various members of the RAS, RAF, MEK, and MAPK/ERK families playing different roles in different contexts. The abovementioned activating mutations in BRAF, NRAS, HRAS, KRAS, KIT, GNAQ, and GNA11 are believed to exert their oncogenic effects, either fully or in part, through the MAPK cascade. The important modulation of this cascade is provided by the additional regulators of the key players. This includes the abovementioned negative regulator of RAS: NF1, which is lost in some cutaneous melanomas [47]. Another notable example is the phosphorylation of both CRAF and MEK1 proteins by PAK1, which reportedly provides a co-stimulatory signal [82,83,84] and facilitates the association between MEK and ERK proteins [85]. Common mutations in RAC1 and PREX2, which eventually activate PAK1, are expected to have the same effect. Mucosal melanomas, which are a very rare type of the disease, have a mutation landscape that is quite diverse and somewhat distinct from those of the cutaneous and uveal forms, but the majority of its cases are still expected to carry an activated MAPK cascade [86,87]. 

Targeting the MAPK cascade has emerged as a standard of care for BRAF-mutant melanoma, where combinations of BRAF and MEK inhibitors have proven to be especially effective at prolonging survival [88,89,90]. Unfortunately, even under a combined therapy, complete responses are achieved in the minority of cases, and are commonly followed by disease recurrence. The responsiveness of NRAS-driven melanomas to targeted therapies is even worse, as BRAF inhibitors are generally inapplicable in this scenario, while MEK inhibitors alone do not offer significant improvements over conventional chemotherapy in the overall survival or the safety profiles [91,92]. Similarly, a MEK inhibitor failed to significantly improve the overall survival in GNAQ- and GNA11-driven uveal melanoma, either when used alone or added to conventional chemotherapy [93,94]. 

The mechanisms of resistance to the inhibitors of the MAPK cascade have received considerable attention. Predictably, activating mutations in NRAS oncogenes provide an alternative pathway to MEK activation, negating the utility of BRAF inhibitors, and are present in therapy-resistant cases [95,96]. Among the earliest recognized resistance mechanisms was also the activation of the PI3K/AKT pathway [41,97,98]. This pathway is long-known to have oncogenic properties [99], and to increase the tolerance to a variety of potentially lethal impacts [100,101,102]. In the context of cancer, the activation of this pathway occasionally may be achieved via activating mutations or the amplification of individual AKT isoforms or, more commonly, via activating mutations in the catalytic subunits of PI3K, via the loss of the tumor suppressor PTEN, or via the activation of receptor tyrosine kinases [96,103] that serve as upstream regulators of PI3K. A number of additional resistance mechanisms have been noted, such as those provided by the Hippo pathway [23] and the kinase PLK3 [104].

PAK1 is an evolutionarily conserved regulator of the MAPK cascade; even in yeast, this cascade is regulated by a PAK1 homologue, Ste20 [105]. Importantly, the role of PAK1 as a co-activator of the MAPK cascade, as well as its close interaction with the PI3K/AKT pathway [106,107], positions PAK1 as a candidate modulator of cell responses to targeted therapies. Singhal et al. discovered that the sensitivity to PAK inhibition distinguishes NRAS- from BRAF-mutant melanomas [108], and this observation was later extended by Ong et al. [43]. The dependence of RAS-mediated transformation on RAC is also known from other systems [50,51,52], but the exact reason for the different drug sensitivity is yet to be firmly established. Of note, it was reported that BRAF, and especially its activated mutant, differ from CRAF based on a reduced dependence on the status of the PAK1 phosphorylation site [84]. Interestingly, it was noted that the inhibition of PAK1 also made NRAS-mutant cells more sensitive to the inhibitors of the MAPK cascade [108], suggesting an avenue to sensitize to targeted therapy this otherwise relatively resistant tumor variant.

The ability of PAK1 to provide additional stimulation to CRAF and MEKs points to a possibility that PAK1 may offer an alternative route for maintaining the minimally required level of ERK activity in BRAF-mutant cells treated with a BRAF inhibitor. Indeed, Babagana et al. demonstrated that in BRAF-mutant cells, the inhibition of PAK1 decreases, while its activation increases the tolerance to BRAF and MEK inhibitors [41]. This phenomenon was later confirmed in an extensive study by Lu et al. [109]. Accordingly, the resistant phenotype can be rendered not only by PAK1 itself, but also by PAK1 activators, such as RAC1 [41,110] and RHOJ [111], and such resistance is negated by PAK1 inhibition. This is in line with the observation that RAC1-driven oncogenesis and metastasis depend on PAK1 [112,113]. Interestingly, the inhibition of PAK1 can also negate the resistance to BRAF inhibitors that is afforded by hyperactive AKT [41]. As mentioned above, AKT is implicated as a key downstream effector of multiple other resistance factors, so the ability to negate its effect potentially expands the utility of PAK1 inhibition as a countermeasure for acquired drug resistance. 

**Figure 3:**
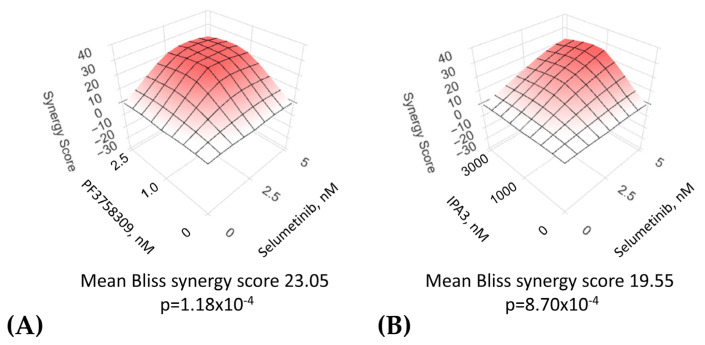
Examples of synergistic suppression of uveal melanoma cells via combinations of PAK inhibitors and a MEK inhibitor, selumetinib. Mel202 uveal melanoma cells [115] (a gift from Dr. Harbour, University of Texas Southwestern) were exposed to a range of doses of selumetinib in combination with various doses of PAK inhibitors PF3758309 (**A**) or IPA3 (**B**). After 5 days of treatment, the numbers of remaining cells were compared using methylene blue staining and an extraction method as described earlier [116], and drug synergy according to the Bliss synergy model was analyzed using SynegyFinder software (version #: 6.04.2023-R-3.6.3-dev) [117].

The recognition of the role of PAK1 in many BRAF wild-type cutaneous melanomas has brought about the question of whether it plays a similar role in the GNA-driven uveal form of the disease [114]. Indeed, as mentioned above, there is evidence that GNA-driven oncogenesis involves the activation of RAS, RAC1, and PAK1. Accordingly, one may predict that, akin to the RAS-driven cases, PAK inhibition in GNA-driven melanomas would enhance the efficacy of targeted therapies. Indeed, a significant synergy between MEK and PAK inhibitors is seen in cultured uveal melanoma cells (Figure 3). Importantly, the synergy is seen even between doses of the drugs that are largely ineffectual when used alone. This provides the hope that these types of drugs, which have not seen a great success in single-drug trials yet, might be more useful in a combined regimen.

It is worth noting that the role of PAK1 in the resistance to targeted therapies is not necessarily limited to its direct effects on the MAPK and AKT pathways. The anti-apoptotic activity of PAK1, including its inhibitory phosphorylation of BAD, may contribute to the phenomenon as well [111]. Moreover, PAK1 has a mutually inhibitory relationship with the tumor suppressor NF2 (aka Merlin) [118,119,120]. The loss of NF2 activity inhibits LATS kinases and consequently relieves from inhibition their target YAP [121], a known determinant of resistance to targeted therapies in melanoma [23].

Overall, ample evidence suggests that PAK1 by itself is a critical vulnerability in a sizeable subset of melanomas, while its co-targeting has the potential to enhance MAPK-directed targeted therapies and circumvent many common modes of resistance to the latter.

## 5. PAK1 and Immunotherapy of Melanoma

The immune system presents a major anti-cancer defense mechanism, but its effectiveness is often attenuated by the immunosuppressive microenvironment created within tumors, as well as the reduced propensity of tumor cells to present their antigens for immunosurveillance. Modern immune therapies most commonly target the “immune checkpoints”, which are exploited by melanoma cells to curtail the activity of cytotoxic T-cells [14,122]. Another type of immunotherapeutic, which to date has shown some efficacy in uveal melanoma, is the ImmTAC (Immune-mobilizing monoclonal T-cell receptor Against Cancer). An ImmTAC acts by tethering T-cells to target cancer cells. A clinically effective [20] ImmTAC, tebentafusp, combines a soluble T-cell receptor that is specific for HLA-bound melanocyte lineage-specific antigen gp100 with an antibody fragment that engages the CD3 receptors on T-cells [123]. Importantly, both the “checkpoint inhibitors” and ImmTAC depend on the successful penetration of T-cells into the tumor and on the presentation of tumor-specific antigens on the surface of tumor cells. The factors that affect these two parameters are expected to affect the efficacy of immunotherapy. This consideration provides some insights into the frequent failure of this therapeutic modality, as well as the avenues to increase its efficacy.

Multiple lines of investigation point to the effects of PAK1 on tumors’ interactions with the immune system and on the various aspects of immunotherapy. Not all of the observations were validated in the context of melanoma, and the relative contribution of various phenomena to the clinical outcomes is yet to be established. The high significance of the topic makes it a very active field of research.

The chemical inhibition or genetic inactivation of PAK reportedly enhanced immunity in a genetically engineered mouse model of intestinal tumorigenesis [124]. Importantly, the absence of PAK1 led to an increase in splenic lymphocytes only in tumor-bearing mice, but not in their tumor-free counterparts, suggesting that the effect is specific for anti-tumor immunity [124]. Interestingly, in this model, the tumor itself had an immunosuppressive effect, which was negated by PAK1 inactivation [124]. The exact mechanism of this phenomenon remains unknown. In part, the role of PAK1 in immune evasion may be expected based on its contribution to the MAPK cascade. Numerous reports suggest that the MAPK cascade contributes to immune evasion in various systems [125,126,127,128] and its inhibition augments immune therapy [129,130,131]. In particular, this pathway appears to enhance the secretion of immunosuppressive cytokines by cancer cells [132] and to limit the ability of cancer cells to present their potential antigens on cell surfaces, while the corresponding inhibitors upregulate HLA class I molecules [125,127]. Intriguingly, a direct interaction was reported between PAK1 and HLA-A, -B, and -C proteins [133], although the biological significance of this phenomenon is still unexplored. Furthermore, the inhibition of the MAPK cascade increases the expression of melanocyte differentiation antigens, which serve as important recognition targets for immunotherapy [126,128]. Thus, the combination of higher-expressed antigens and more effective antigen presentation could make tumors a better target for the immune system. Interestingly, the MAPK cascade in melanoma cells also contributes to the production of IL-1, which stimulates the release of immunosuppressive secreted factors by tumor-associate fibroblasts, thus contributing to the immunosuppressive tumor microenvironment [134]. Furthermore, it was reported that a melanoma-derived mutant RAC1 (RAC1 P29S) selectively drives the expression of PD-L1 [135], a major immune-evasive molecule [136]. However, the molecular mechanism of this phenomenon remains unexplored, and it is peculiar that the effect was limited to this, but not the other (RAC1 Q61L) constitutively active RAC1 variant [135]. Thus, it is uncertain whether PAK1 mediates the control of PD-L1 by RAC1 in that system. However, the role of PAK1 in PD-L1 control has been shown more convincingly in pancreatic cancer models, where the loss of PAK1 decreased PD-L1 expression while increasing tumor infiltration by CD4+ and CD8+ T-cells [137].

The expression of FasL by melanoma cells was proposed as one of the mechanisms that protects the tumors from the immune system by killing vulnerable T-cells [138,139]. The actual incidence of FasL expression in melanomas is a subject of a controversy [140], but it is noteworthy that FasL is also produced by the endothelial cells in the tumor [141]. Interestingly, the death of T-cells from FasL depends on the function of RAC proteins [142]. It is thus possible that the inhibition of RAC signaling would improve anti-tumor immunity and the efficacy of immune therapy, but the role of PAK1 in FasL-mediated killing is yet to be established.

Importantly, the systemic inhibition of the MAPK cascade is also known to affect the immune system directly, wherein it is expected to enhance the anti-tumor response. This is especially relevant to MEK inhibitors, which can suppress B-regulatory cells [143] and increase the number of effector-phenotype antigen-specific CD8-positive T-cells within the tumor [129]. These inhibitors also extend the life of tumor-infiltrating T-cells, while sparing their cytotoxic activity [129]. Overall, the efficacy of immune therapy in preclinical models is enhanced by a more efficient inhibition of the MAPK cascade [125,126,127,129]. It is therefore likely that further suppression of this pathway via a concomitant inhibition of PAK1 would be beneficial for immune therapy. 

An obvious concern is whether some MAPK-independent functions of PAK1 are critically essential for the immune system, so that PAK1 inhibition would be immunosuppressive. The observations in the PAK1-deficient mouse model seem to dispel this concern, as the knockout animals demonstrated heightened, rather than decreased, immunity [124,137]. This supports the enthusiasm for a further investigation of PAK1 inhibition as an auxiliary for immune therapy.

## 6. Future Directions

While the preponderance of evidence points to PAK1 inhibition as a promising strategy to augment various types of anti-melanoma therapies, the need for a clinically usable approach to PAK1 regulation remains unmet. To date, there is a multitude of chemical agents that can inhibit this enzyme, yet none have received an approval for clinical use. The histories, chemical structures, specificities, and investigational status of these molecules have been extensively reviewed by others elsewhere [39,40,144]. The hurdles for the currently available compounds range from chemical instability and poor bioavailability to off-target effects and expected toxicity. Several directions forward can be envisioned. The development of new compounds remains a possibility. A critical issue on this path remains to be the balance between isoform specificity and anti-cancer activity. PAK1-deficient animals have a relatively mild phenotype [145], although they are prone to cardiac problems under specific types of stress [146]. Non-transformed cells have a higher tolerance to PAK1 inhibition [50], and may even benefit from it under certain conditions [147]. In contrast, PAK2 deficiency is embryonically lethal [148,149], while PAK3-deificent mice display deficits in some memory- and learning-related tasks [150]. Concededly, the phenotypes of the knockout animals may only imperfectly recapitulate the effects of systemic target inhibition in an adult organism. Nevertheless, it is reasonable to assume that a PAK1-specific inhibitor would be less prone to off-target toxicity than the one targeting all group 1 PAKs, not to mention the inhibitors with even broader specificity. Toxic side effects may be further compounded by the spurious inhibition of more distantly related enzymes. A noteworthy recent development [151] is the report of a PAK1-specific degrader molecule (BJG-05-039), which combines a highly selective, but only modestly potent, PAK1 inhibitor, NVS-PAK1-1 [152] with lenalidomide, a recruiter of the CRL4^CRBN^ E3 ubiquitin ligase [153]. The approach results in a significant reduction of PAK1 protein levels in treated cells, and potentially provides a concurrent suppression of the enzymatic and the scaffolding functions of PAK1 [151]. The high specificity and potency of BJG-05-039 against PAK1 suggest an opportunity to avoid the off-target toxicity of less specific PAK inhibitors. On the other hand, there is some functional overlap between PAKs, which brings up the possibility that a treated cancer may evolve a resistance to complete PAK1 inhibition by elevating the activity of other isoforms [49]. Thus, a broader-specificity inhibitor might be needed for a more robust anti-cancer effect. 

Conceivably, a practical resolution might emerge from the use of PAK inhibitors not as monotherapies, but in synergistic combinations with other anti-cancer treatments. In general, the search for a synergistic drug combination for melanoma treatment remains an active area of research [154]. Some examples of combinations, where the synergy of other therapeutics with PAK1 inhibitors is documented or may be predicted, are discussed above. Each component in such a combination may have some preferential toxicity to cancer cells, albeit with some dose-dependent side effects. In an ideal case, synergy would allow the combination to achieve a therapeutic effect even when the individual components are delivered at doses that are safe but ineffective upon individual administration, i.e., when the respective targets are only partially inhibited. In this scenario, even an inhibitor with otherwise non-ideal pharmacokinetics or pharmacodynamics might prove useful, as long as it potently synergizes with other components of the combination regimen. 

Another possible avenue for drug improvement is the optimization of the delivery options for the existing compounds. For example, IPA3, a pioneering compound that selectively disrupts the interaction between group 1 PAKs and small GTPases [155], was considered to be sub-optimal for clinical use due to its perceived metabolically labile nature [156]. However, more recent studies suggest that at least some of its shortfalls can be overcome by formulating it in liposomes [157,158]. 

It is also worth mentioning that PAK1 can be regulated indirectly by preventing its activation by cancer-relevant small GTPases. Targeting a particular cancer-relevant GTPase may prevent the pathological activation of a PAK enzyme, while preserving at least some of its normal physiological functions. The inhibition of small GTPases is a burgeoning area of pharmacological research. For example, there are already a number of potent and relatively specific inhibitors of RAC1 [159], the eponymous activator of PAK1. However, the hurdles in the clinical translation of these discoveries still remain [159], and none have been approved as an anti-melanoma therapy. Previously, the research into the regulation of small GTPases was mostly focused on their interactions with guanine nucleotide exchange factors, guanine nucleotide dissociation inhibitors, and GTPase-activating proteins [160]. Recent findings suggest that the activity of RAC1 can also be manipulated by controlling the local availability of GTP [161]. The clinical effectiveness of this strategy is yet to be proven, but multiple inhibitors of GTP biosynthesis are already approved for human use. 

Another approach, yet to be implemented in practice, is to exploit the higher activity of PAK1 in treatment-resistant cells as a source of a specific vulnerability. One possible line of exploration is the intracellular stress of the hyperactivation of PAK1. The hyperactivity of oncogenic pathways is often damaging to a cell, making it exceptionally dependent on cytoprotective stress-response pathways and sensitive to further impacts that elevate the same type of damage. Paradoxically, this scenario applies even to oncogenes that are otherwise known to protect cancer cells from common therapies, as exemplified by AKT [116,162]. Another way of exploiting PAK1 activation may be to rely on its effect on DNA damage response. While its ability to impair damage sensing mechanisms may increase cell tolerance to individual genotoxic compounds [73], the failure of cell cycle checkpoints may also sensitize a cell to conditions which a normal cell can survive by arresting its cell cycle progression. Indeed, such strategies to exploit cell cycle checkpoint deficiencies, albeit not in a PAK1-dependent manner, have been proposed for melanoma already [163,164]. 

Overall, the available information on the functions of PAK1, the experimental data from preclinical melanoma models, and the evidence from similarly treated non-melanoma cancers all point to PAK1 as a modulator of melanoma’s response to multiple types of anti-cancer therapy. The co-targeting of PAK1 in the context of other melanoma treatments appears as a worthwhile direction for future exploration, with a potential to tangibly improve the management of this deadly malignancy. 

## Data Availability

The data presented in this study are available on request from the corresponding author.
